# It All Depends What You Count—The Importance of Definitions in Evaluation of CF Screening Performance

**DOI:** 10.3390/ijns6020047

**Published:** 2020-06-10

**Authors:** Natasha Heather, Dianne Webster

**Affiliations:** 1 National Newborn Metabolic Screening programme, LabPlus, Auckland City Hospital, Auckland 1148, New Zealand; diannew@adhb.govt.nz; 2 Liggins Institute, University of Auckland, Auckland 1023, New Zealand

**Keywords:** newborn screen, target disorder, missed case, sensitivity, cystic fibrosis, CFSPID, immunoreactive trypsin, meconium ileus

## Abstract

Screening metrics are essential to both quality assessment and improvement, but are highly dependent on the way positive tests and cases are counted. In cystic fibrosis (CF) screening, key factors include how mild cases of late-presenting CF and CF screen positive, inconclusive diagnosis (CFSPID) are counted, whether those at prior increased risk of CF are excluded from the screened population, and which aspects of the screening pathway are considered. This paper draws on the New Zealand experience of almost forty years of newborn screening for CF. We demonstrate how different definitions impact the calculation of screening sensitivity. We suggest that, to enable meaningful comparison, CF screening reports should clarify what steps in the screening pathway are included in the assessment, as well as the algorithm used and screening target.

## 1. Introduction

Most newborn screening programmes want to know how they are performing. Local metrics, such as transit times for samples or the efficiency of short-term followup of unsuitable samples, are influenced by local conditions and can usefully be compared from time to time within a programme. Global metrics such as those used in public health (e.g., screening sensitivity, specificity, and positive predictive value) are widely used to compare performance between programmes. However, the comparison may not be based on equivalent counting of positive tests and detected and missed cases. This article explores the different definitions used in cystic fibrosis (CF) screening and the effects on screening metrics.

## 2. Factors to Consider

### 2.1. Target Disorder

When newborn screening started in the 1960s the understanding of disease was simpler—a baby either had PKU or not. As time went by it was recognized that a milder form existed and the baby had PKU or hyperphenylalaninemia. Then it became clear the borders between these conditions were not sharp, and considerable effort (phenylalanine loads) went into deciding whether a baby with a raised phenylalanine level had hyperphenylalaninemia type one to five (from benign to severe). Finally, the spectrum of disease was recognized, and now it is considered that each person with raised blood phenylalanine has their own disease determined not only by variants in the phenylalanine hydroxylase gene but also by other protein-metabolizing and amino acid-transporting systems.

Similarly, at the time that screening for CF started in the late 1970s [1] it was considered to be a uniformly serious childhood condition. However, since the discovery of the cystic fibrosis transmembrane regulator (CFTR) [2], the CF phenotype has been broadened to include mild and late-presenting disease, such as otherwise healthy males presenting with infertility and older adults with mild respiratory symptoms but found to carry two “pathogenic” CFTR variants [3]. Many CF screening programmes have only been in place for a few years. The recognition of a broadened CF phenotype has created problems in defining the outcome as well as the target of screening.

The biological level at which screening and confirmatory investigations are performed (genetic and/or functional assessment) impacts the number and severity of cases that will be detected [4]. The 2017 CF Foundation consensus guidelines reinforce the importance of a sweat test in establishing a diagnosis of CF [5]. The NewSTEPS case definition acknowledges that a sweat test is the gold standard, but accepts that a diagnosis could also be established by genotyping [6]. Furthermore, most screening programmes would say that a detected case is an infant with a positive newborn screen who went on to be diagnosed with CF. However, some infants have ambiguous genotypes (e.g., one pathogenic variant and another variant of unknown significance) and/or biochemical phenotypes (low but still abnormal sweat chlorides, such as 30–59 mmol/L) and may or may not develop classical CF symptoms later. These infants are now described as CF screen positive, inconclusive diagnosis (CFSPID) [7,8].

This raises the question—what is a diagnosis? When an infant presents with meconium ileus or failure to thrive, the diagnosis is CF. When an infant has a positive screen, confirmatory tests (sweat and pancreatic function) and possibly previously unidentified clinical features can also lead to the early diagnosis of CF. However, infants with CFSPID are apparently healthy, asymptomatic infants who are essentially diagnosed based on their newborn screen, as further tests have been inconclusive. CFSPID sounds like a disease, which creates anxiety and confusion for families [9]. Yet, such infants may go on to either develop symptoms of CF or remain healthy. Screening and sometimes confirmatory investigations provide an indication of the risk of disease [4]. In newborn screening, post-analytical tools, such as the Collaborative Laboratory Integrated Report (CLIR), are being developed to assist with such assessments of risk [10]. It may be that the outcomes of screening could be CF confirmed, CF remains possible, CF unlikely—and results communicated to families in that way.

Screening metrics are used for programme evaluation and to inform quality improvements. Whilst some programmes aim to detect all possible cases, others apply pragmatic boundaries to missed cases such as those presenting in early childhood with severe disease. It is not clear from the literature whether different programmes consider CFSPID as cases of screen-detected CF, and we think it likely that CFSPID is sometimes counted and sometimes not. Whatever approach is taken, the case definition should be clear and consistent, as it impacts screening metrics. In order to inform quality improvements, outcome data must also be available within a reasonable timeframe. The benefit of knowing about a case missed more than a decade prior is arguable given likely changes to the test methodology and algorithms in the intervening period.

### 2.2. Screened Population

Definitions of population screening vary but generally include a statement about screening only being appropriate for persons not at increased prior risk of having the disorder [11]. The argument for this is that at-risk infants, such as those with a family history of CF, should have genetic and functional diagnostic testing performed regardless of the newborn screen result (with genetic testing taking the particular family CFTR variants into account).

The impact of including at-risk infants in screening metrics varies depending on the screening algorithm used, and hence what is defined as a positive test.

- If the first step of the algorithm is whether family history or meconium ileus is present, and all are reported as positive screens, then all CF cases within this high-risk group will be counted as detected by screening.
- If the first step of the algorithm is to measure immunoreactive trypsin (IRT), then only those with raised IRT will be reported as screen positive, and those who have a family history but do not have raised IRT (as is common in severe disease, especially with meconium ileus detected [12]) will be counted as missed cases.
- If, following a raised IRT, the second step of the algorithm is CFTR variant analysis using a common CFTR variant panel, the screen will also miss those with family histories and a raised IRT but uncommon CFTR variants that are not included in the panel used.

### 2.3. Programme Boundaries

When calculating screening metrics, jurisdictions apply variable boundaries to the screening programme. Many jurisdictions only count missed cases if a normal screen result was issued. As a result, the count of missed cases is limited to those occurring within the laboratory, and due to either screening protocols or error. Whilst this definition may focus on aspects under the control of the screening laboratory, it will result in fewer missed cases and higher reported sensitivity than jurisdictions which apply a broader definition of missed cases.

CF can be missed at all steps of the screening and diagnostic pathway, including where no screening occurred or during the short-term followup [13,14]. Some jurisdictions count missed cases that occur early in the screening pathway because either the test is not offered or the family declines. Others consider the screening pathway to begin with the acceptance of a screening offer, and so would not count cases where families have declined screening because the family has effectively removed itself from the screened population. Cases may also be missed at the level of short-term followup, because the appropriate followup did not occur, or because the diagnostic test was either misinterpreted or incorrectly performed. This is particularly relevant to CF, as both methodological and biological variation can impact measured sweat chloride [15].

### 2.4. CF Screening Sensitivity Example

Newborn screening for CF by the measurement of IRT in dried blood spots was developed in New Zealand [1], and this was the first national programme to adopt CF screening in 1981 [16]. The programme now follows a two-step algorithm whereby samples with raised IRT (top 1%) reflex to analysis for common CFTR variants (F508del, G542X, G551D and in later years R117H). Aside from the addition of R117H, the algorithm remained the same over the period reported. The ethnic composition of New Zealand births has changed over the past decades [17] and was recently described for the period 2010–2017 [18].

Those with at least one CFTR variant are reported as positive CF screening tests. All positive tests within the Auckland region are referred to the multidisciplinary CF team at Starship Children’s Hospital, who are also referred likely cases of CF from community and hospital teams within the region. We utilized the Starship Children’s Hospital CF clinical database to identify new CF cases and to review CF screening in the Auckland region between 2003 and 2017.

In this time period, 325,000 babies were screened. There were 113 cases of CF diagnosed, of whom 89 were diagnosed as a result of positive newborn screening tests and 24 were clinically detected. Eight CF cases were excluded from further analysis as they had been born abroad and not screened in New Zealand. Of note, seven of these had not been screened for CF and one had a positive screen followed by a sweat test result that was considered to be normal. Table 1 outlines the relevant screening factors for the 16 New Zealand-born CF cases that were diagnosed clinically.

The calculation of screening sensitivity (the number of true positive screens divided by the sum of true positive and false negative screens, expressed as a percentage) varies depending on which clinically diagnosed CF cases are included in the count of missed cases.

- If sensitivity is calculated by counting all missed cases: 89/105 = 84.8%
- If the sensitivity calculation does not include those outside the screened population (i.e., screening declined, family history or meconium ileus) as missed cases: 89/102 = 87.3%
- If the sensitivity calculation only counts missed cases as those which occurred within the laboratory (in-range IRT, no CFTR variants on panel): 89/100 = 89.0%

## 3. Conclusions

While screening metrics are essential for both the quality assessment and improvement of programmes, they are highly dependent on the way positive tests and cases are counted. It is difficult to compare programme metrics unless definitions of the target disorder, the screened population, and the screening programme boundaries are clear and constant over time. This is particularly true for CF, where screening algorithms vary and there is a broad phenotype, as well as infants labelled with CFSPID. We suggest that in order to enable the meaningful comparison of performance data, CF screening reports should clarify what steps in the screening pathway are included in the assessment, as well as the algorithm used and screening target.

## Figures and Tables

**Table 1:** Clinically diagnosed cystic fibrosis, Auckland region 2003–2017.

	No Other Factors	Other Factors	Screening Boundary	Total
No screen			1 declined	1
In-range IRT ^†^	9	1 MI ^††^		10
CFTR variant not on panel	2	1 FH ^†††^		3
Positive screen			2 normal sweat test	2
Total				16

^†^ IRT = immunoreactive trypsin; ^††^ MI = meconium ileus; ^†††^ FH = family history.

## References

[1] Crossley J.R., Elliott R.B., Smith A. (1979). Dried-blood spot screening for cystic fibrosis in the newborn. Lancet.

[2] Kerem B., Rommens J.M., Buchanan J.A., Markiewicz D., Cox T.K., Chakravarti A., Buchwald M., Tsui L.C. (1989). Identification of the cystic fibrosis gene: Genetic analysis. Science.

[3] Gilljam M. (2004). Clinical manifestations of cystic fibrosis among patients with diagnosis in adulthood. Chest.

[4] Pollitt R.J. (2015). Different viewpoint: International perspectives on newborn screening. J. Med. Biochem..

[5] Farrell P.M., White T.B., Ren C.L., Hempstead S.E., Accurso F., Derichs N., Howenstine M., McColley S.A., Rock M., Rosenfeld M. (2017). Diagnosis of Cystic Fibrosis: Consensus Guidelines from the Cystic Fibrosis Foundation. J. Pediatr..

[6] Sontag M.K., Sarkar D., Comeau A.M., Hassell K., Botto L.D., Parad R., Rose S.R., Wintergerst K.A., Smith-Whitley K., Singh S. (2018). Case definitions for conditions identified by newborn screening public health surveillance. Int. J. Neonatal Screen.

[7] Munck A., Mayell S.J., Winters V., Shawcross A., Derichs N., Parad R. (2015). Cystic Fibrosis Screen Positive, Inconclusive Diagnosis (CFSPID): A new designation and management recommendations for infants with an inconclusive diagnosis following newborn screening. J. Cyst. Fibros..

[8] Levy H., Farrell M. (2015). New challenges in the diagnosis and management of cystic fibrosis. J. Pediatr..

[9] Johnson F., Southern K.W., Ulph F. (2019). Psychological impact on parents of an inconclusive diagnosis following newborn bloodspot screening for cystic fibrosis: A qualitative study. Int. J. Neonatal Screen.

[10] Collaborative Laboratory Integrated Reports. https://clir.mayo.edu.

[11] National Health Committee (2003). Screening to Improve Health in New Zealand.

[12] Sontag M.K., Corey M., Hokanson J.E., Marshall J.A., Sommer S.S., Zerbe G.O., Accurso F.J. (2006). Genetic and physiologic correlates of longitudinal immunoreactive trypsinogen decline in infants with cystic fibrosis identified through newborn screening. J. Pediatr..

[13] Holtzman C., Slazyk W.E., Cordero J.F., Hannon W.H. (1986). Descriptive epidemiology of missed cases of phenylketonuria and congenital hypothyroidism. Pediatrics.

[14] Henry R.L., Boulton T.J., Roddick L.G. (1990). False negative results on newborn screening for cystic fibrosis. J. Paediatr. Child Health.

[15] Collie J.T., Massie R.J., Jones O.A., LeGrys V.A., Greaves R.F. (2014). Sixty-five years since the New York heat wave: Advances in sweat testing for cystic fibrosis. Pediatr. Pulmonol..

[16] Wesley A.W., Smith A., Elliott R.B. (1989). Experience with neonatal screening for cystic fibrosis in New Zealand using measurement of immunoreactive trypsinogen. Aust. Paediatr. J..

[17] Albert B.B., Cutfield W.S., Webster D., Carll J., Derraik J.G.B., Jefferies C., Gunn A.J., Hofman P.L. (2012). Etiology of increasing incidence of congenital hypothyroidism in New Zealand from 1993–2010. J. Clin. Endocrinol. Metab..

[18] Heather N.L., Derraik J.G., Webster D., Hofman P.L. (2019). The impact of demographic factors on newborn TSH levels and congenital hypothyroidism screening. Clin. Endocrinol. (Oxf.).

